# Inhibition of PI3K/AKT signaling via ROS regulation is involved in Rhein-induced apoptosis and enhancement of oxaliplatin sensitivity in pancreatic cancer cells

**DOI:** 10.7150/ijbs.49514

**Published:** 2021-01-15

**Authors:** Yuhui Liu, Chengjian Shi, Zheng He, Feng Zhu, Min Wang, Ruizhi He, Chunle Zhao, Xiuhui Shi, Min Zhou, Shutao Pan, Yang Gao, Xu Li, Renyi Qin

**Affiliations:** 1 Department of Biliary-Pancreatic Surgery, Affiliated Tongji Hospital, Tongji Medical College, Huazhong University of Science and Technology, Wuhan, Hubei, China.; 2 Department of General Surgery, Shiyan People's Hospital of Bao'an Distict, Shenzhen, Guangdong, China.

**Keywords:** Pancreatic cancer, Rhein, PI3K/AKT pathway, Reactive oxygen species, Apoptosis, Oxaliplatin

## Abstract

Several natural products have been demonstrated to both enhance the anti-tumor efficacy and alleviate the side effects of conventional chemotherapy drugs. Rhein, a main constituent of the Chinese herb rhubarb, has been shown to induce apoptosis in various cancer types. However, the exact pharmacological mechanisms controlling the influence of Rhein on chemotherapy drug effects in pancreatic cancer (PC) remain largely undefined. In this study, we found that Rhein inhibited the growth and proliferation of PC cells through G1 phase cell cycle arrest. Moreover, Rhein induced caspase-dependent mitochondrial apoptosis of PC cells through inactivation of the PI3K/AKT pathway. Combination treatment of Rhein and oxaliplatin synergistically enhanced apoptosis of PC cells through increased generation of intracellular reactive oxygen species (ROS) and inactivation of the PI3K/AKT pathway. Pre-treatment with the ROS scavenger N-acetyl-L-cysteine attenuated the combined treatment-induced apoptosis and restored the level of phosphorylated AKT, indicating that ROS is an upstream regulator of the PI3K/AKT pathway. The combination therapy also exhibited stronger anti-tumor effects compared with single drug treatments *in vivo*. Taken together, these data demonstrate that Rhein can induce apoptosis and enhance the oxaliplatin sensitivity of PC cells, suggesting that Rhein may be an effective strategy to overcome drug resistance in the chemotherapeutic treatment of PC.

## Introduction

Pancreatic cancer (PC) is a highly malignant cancer that has become the fourth leading cause of cancer-related death in men and women in US 1. Although progress has been made in the treatment of PC in recent years, the 5-year survival rate of this disease remains less than 9% 2. Because of a lack of symptoms, patients are often diagnosed with PC at an advanced stage. Only a few patients have an opportunity for surgical resection, which makes chemotherapy an important component in the systemic treatment of PC 3, 4.

The first-line chemotherapeutic regimen for PC includes gemcitabine, 5-fluorouracil, and platinum-based drugs 5, 6. Traditional chemotherapy drugs function mainly by interfering with DNA synthesis, restraining mitotic processes, and inducing apoptosis, eventually leading to cancer cell death 7, 8. However, cancer cells can sometimes develop resistance to these drugs through various mechanisms such as changes in DNA repair machinery, overexpression of drug efflux pumps, and activation of anti-apoptotic genes and pathways 9, 10. Therefore, drugs that enhance the anti-cancer effects of existing chemotherapy regimens need to be developed.

**Figure 1:**
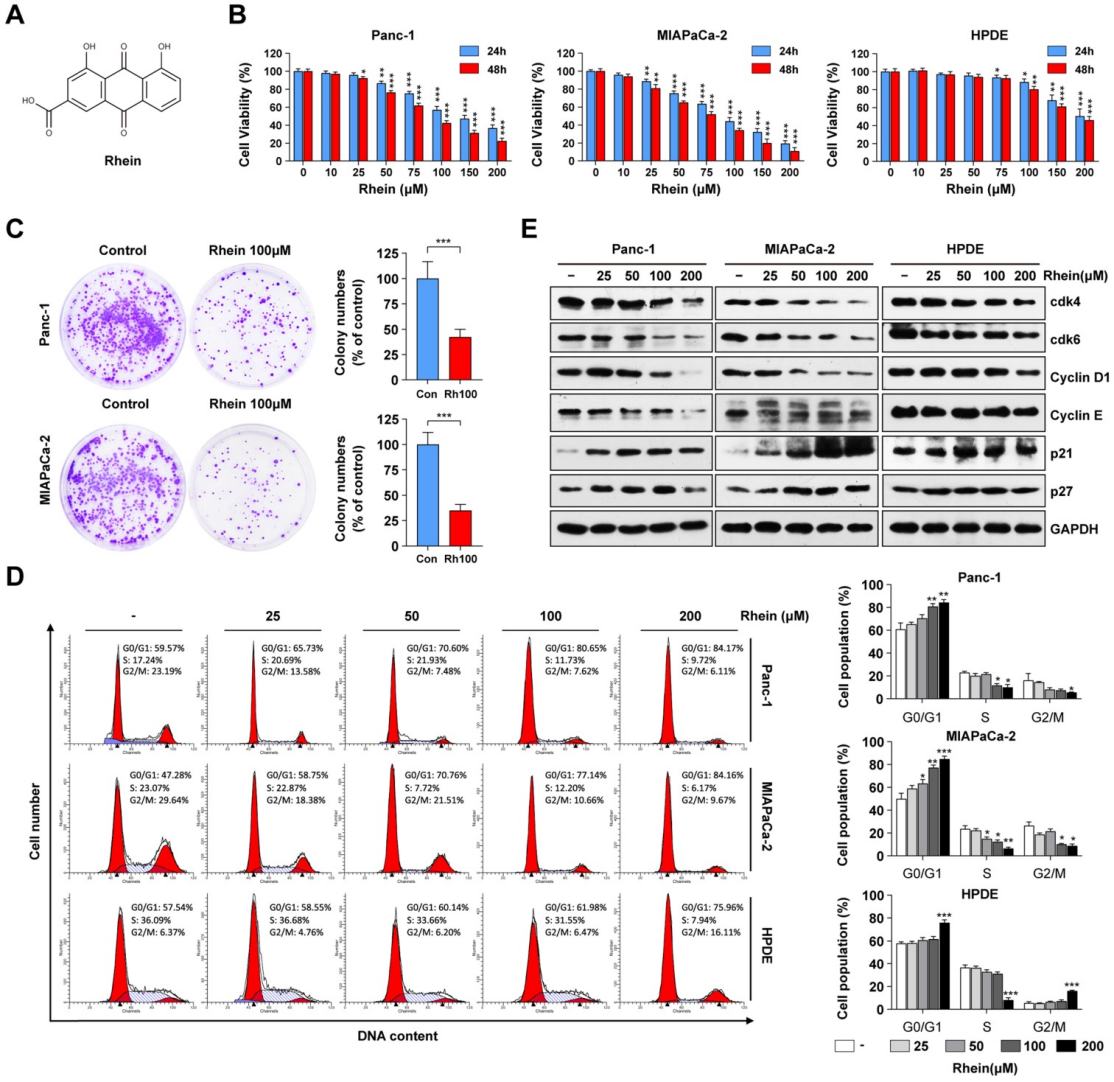
**Rhein inhibits PC cell growth by inducing G1 phase cell cycle arrest.** (A) The chemical structure of Rhein. (B) Panc-1, MIAPaCa-2 and HPDE cells were treated with 0-200 μM of Rhein for 24 or 48 h. CCK-8 assays were performed to determine the cell viability. *p < 0.05; **p < 0.01; ***p < 0.001. (C) Panc-1 and MIAPaCa-2 cells were cultured for 14 days to form colonies after treated with 100 μM Rhein or DMSO for 24 h. Representative images are shown. Each bar represents means± SD from three independent experiments. ***p < 0.001. (D) Cell cycle distribution were analyzed in Panc-1, MIAPaCa-2 and HPDE cells after 0-200 μM Rhein treatment. Representative results are shown in the left panel. Statistical comparisons were performed. Each bar represents means ± SD from three independent experiments. *p < 0.05; **p < 0.01; ***p < 0.001. (E) Western blot analysis of protein levels of cdk4, cdk6, cyclinD1, cyclinE, p21 and p27 in Panc-1, MIAPaCa-2 and HPDE cells after indicated concentration of Rhein treatment for 24 h. GAPDH was used as a loading control. Representative results of two independent experiments are shown.

Rhein (4, 5-dihydroxyanthraquinone-2-carboxylic acid) (Figure 1A), an anthraquinone compound extracted from the traditional Chinese herb rhubarb, has been used for centuries for the treatment of diabetic nephropathy and inflammatory diseases 11. Recent studies have demonstrated the anti-inflammatory and anti-virus activities of Rhein at a concentration 12, 13. Rhein can also inhibit the proliferation and induce apoptosis of renal and colorectal cancer cells by inducing cell cycle arrest and affecting various signaling cascades, including the STAT3, mitogen-activated protein kinase (MAPK), nuclear factor-kappa B (NF-κB), and apoptotic pathways^14^, ^15^. It is reported that Rhein has an inhibitory effect on the PI3K/AKT pathway in breast and lung cancers 16, 17. In PC, Rhein can inhibit hypoxia-inducible factor-1α (HIF-1α) and suppress cell growth 18. Another study revealed that Rhein inhibits the STAT3 pathway and sensitizes PC cells to EGFR inhibitors 19. However, the influence of Rhein on the PI3K/AKT pathway in PC has not been reported. Additionally, the exact pharmacological mechanisms controlling the influence of Rhein on chemotherapy drug effects in PC remain largely undefined.

In the present study, we investigated the effects of Rhein treatment alone or in combination with oxaliplatin on PC cells. We demonstrated for the first time that Rhein can inhibit the PI3K/AKT pathway and display a synergistic effect with oxaliplatin by inducing apoptosis of PC cells both *in vitro* and *in vivo*. These results may provide a novel combination chemotherapy strategy for the treatment of PC.

## Materials and Methods

### Antibodies and reagents

The following antibodies and reagents were purchased: CDK4 (#12790), CDK6 (#13331), cyclin D1 (#55506), cyclin E (#20808), p21 (#2947), p27 (#3686), PARP (#9532), caspase-9 (#9504), cleaved-caspase-3 (#9664), AKT (#4691), phospho-AKT (Ser473) (#4060), phospho-PDK1 (Ser241) (#3438), PTEN (#9188), phospho-PTEN (Ser380) (#9551) and phospho-c-raf (Ser259) (#9421) antibodies were purchased from Cell Signaling Technology (Beverly, MA, USA). Bcl-2 (ab32124), Bcl-XL (ab32370), Bax (ab32503) and cytochrome c (ab133504) antibodies were purchased from abcam. GAPDH (60004-1-Ig), Survivin (10508-1-AP), XIAP (66800-1-Ig), COXIV (11242-1-AP) and PCNA (60097-1-Ig) antibodies were purchased from Proteintech Group (Chicago, IL, USA). Rhein (R7269), Oxaliplatin (O9512) and N-acetyl-L-cysteine (NAC, A7250) were obtained from Sigma-Aldrich (St. Louis, MO, USA). DCFH-DA (S0033) and JC-1 (C2006) were purchased from Beyotime (Haimen, China). Z-DEVD-FMK (S7312), z-LEHD-FMK (S7313) and z-VAD-FMK (S8102) were purchased from Selleck (Houston, TX, USA). LY294002 (HY-10108) and 740Y-P (HY-P0175) were purchased from MCE (Monmouth, NJ, USA).

### Cell lines and culture

Human pancreatic cancer cell lines (Panc-1 and MIAPaca-2) were purchased from American Type Culture Collection (Manassas, VA, USA). The Immortalized human pancreatic ductal epithelial cell HPDE was obtained from Beijing North Carolina Chuanglian Biotechnology Research Institute (Beijing, China). Cells were cultured in Dulbecco's modified Eagle's medium (Gibco, NY, USA) supplemented with 10% fetal bovine serum (FBS) and 100 U/ml of penicillin/streptomycin. Cells were maintained in a humidified incubator at 37 ℃ with 5% CO_2_.

### Cell viability assay

Cell viability was assessed with the Cell Counting Kit-8 (CCK-8) assay (Dojindo Molecular Technologies, Kumamoto, Japan) according to manufacturer's instructions. In brief, 3*10^3^ cells in 100 μl culture medium were plated in a 96-well plate. After adherence, cells were treated with reagents for certain time as indicated by the figures. Subsequently, culture media were replaced and 10 μl CCK-8 solution were added to each well and incubated in 37 ℃ for 1 h. Absorbance at 450nm was measured using a microplate reader (Biotek Instruments, USA). To investigate the combined effect of oxaliplatin and Rhein, cells were treated with different ratio of drug concentrations and combination index (CI) was calculated using CalcuSyn software (Biosoft, Ferguson, MO, USA).

### Colony formation assay

To explore the long-term effects of drug treatment, 1*10^3^ cells were seeded in 60 mm dish. After adherence, cells were treated with drugs for indicated concentrations for 24 h. Media were replaced by complete cell cultural medium without drug every 2-5 days. On day 14, cells were washed with PBS twice, fixed with 4% paraformaldehyde for 30 min and stained by 0.1% crystal violet for 30 min. Colonies were then photographed and counted.

### Cell cycle analysis and hypodiploid cell population determination

After treated with indicated drugs, cells were harvested and washed with PBS before fixing with cold ethanol (70% v/v) at 4℃ for 24 h. Cells were then washed, resuspended with cold PBS and 20 μl RNase A (50 μg/ml) were added and incubated at 37℃ for 30 min. 20 μl propidium iodide (PI) (50 μg/ml) were added and incubated in dark at 4℃ for 30 min. Distribution of cells with different DNA content or hypodiploid (sub-G1) cell populations which indicated apoptosis were then analyzed by flow cytometry on FACS Calibur flow cytometer (BD Immunocytometry Systems, USA).

### Annexin V and PI staining

Cell apoptosis were detected by Annexin V-FITC/PI apoptosis kit (AP101, MultiSciences Biotech Co. Ltd, China). In brief, about 1*10^5^ cells per sample were harvested after indicated treatment and washed with PBS twice. Each sample was resuspended with 500 μl 1× binding buffer and incubated with 5 μl Annexin V and 10 μl PI for 15 min at room temperature in dark. Samples were then analyzed by flow cytometry on FACS Calibur flow cytometer (BD Immunocytometry Systems, USA).

### Mitochondrial transmembrane potential assessment

The fluorescent dye JC-1 was used to detect mitochondrial transmembrane potential (Δ*ψ*m). After treated with indicated drugs, cells were harvested and washed with PBS and incubated with fresh medium containing JC-1 at 37℃ for 20 min. Cells were then washed with staining buffer twice and subjected to flow cytometry.

### Detection of intracellular reactive oxygen species

The intracellular reactive oxygen species (ROS) was measured by 2, 7-dichlorofluorescein diacetate (DCFH-DA) staining. After indicated treatment, cells were harvested and washed with PBS. The cells were incubated with 10 μM DCFH-DA in Hank's balanced salt solution (HBSS) at 37℃ for 20 min. Cells were washed twice, resuspended with PBS and subjected to flow cytometry.

### Protein isolation and Western blot analysis

Cells or tissues were lysed in RIPA buffer (Boster Biological Technology, Wuhan, China) containing the protease inhibitor cOmplete and PhosSTOP (Roche, IN, USA). After sonication and centrifugation, supernatant was collected and protein concentration was measured using BCA Protein Assay Kit (Beyotime). Equal amounts of protein were loaded and separated by SDS-PAGE and then transferred onto polyvinylidene difluoride (PVDF) membranes (Millipore, MA, USA). Target antigens were incubated with specific primary antibodies (1:500-1:1000 dilution) at 4℃ overnight followed by horseradish peroxidase-conjugated secondary antibodies at room temperature for 1h. Target bands were then developed using an enhanced chemiluminescent horseradish peroxidase substrate (Thermo Fisher Scientific, MA, USA). For the detection of translocation of cytochrome C and Bax, we used the Cell Mitochondria Isolation Kit (Beyotime) to separate mitochondrial and cytoplasmic protein according to manufacturer's instructions and then performed Western blot analysis as mentioned above.

### Xenograft experiments

Animal studies were approved by The Institutional Animal Care and Treatment Committee of Huazhong University of Science and Technology. Male nude BALB/c mice at 6 to 8 weeks old were obtained from HFK BioTechnology (Beijing, China). Panc-1 cells (2*10^6^ cells per mouse) were mixed with Matrigel (Becton Dickinson, CA, USA) at a ratio of 1:1 and were injected subcutaneously into the mice. When the tumor had reached a volume of about 100mm^3^, mice were randomized into 4 groups (n=5) and given intraperitoneal injection 3 times a week as following: vehicle alone (10% DMSO, 40% Cremophor/ethanol (3:1), and 50% PBS), oxaliplatin alone (10 mg/kg), Rhein alone (50 mg/kg) or a combination of oxaliplatin (10 mg/kg) and Rhein (50 mg/kg). Body weight and tumor size of the mice were measured twice a week from week 3 after implantation. Tumor volume were calculated as V =1/2 * (length * width^2^). At the end of treatments, all mice were sacrificed and tumors were obtained, weighted and embedded in paraffin for further analysis.

**Immunohistochemistry and TUNEL assay**

The collected tumor samples were embedded in paraffin, cut into 4-μm sections and subjected to H&E staining or incubated with proliferating cell nuclear antigen (PCNA, 1:200 dilution) antibody for immunohistochemistry. TUNEL assay were performed using an ApopTag Peroxidase In Situ Apoptosis Detection Kit (Roche, S7100) according to manufacturer's instructions.

### Malondialdehyde (MDA) assay

MDA detection kit (Beyotime Biotechnology, Nantong, China) was used to detect the MDA level of tumor xenograft. In brief, protein of tumor xenograft was isolated with RIPA buffer. After sonication and centrifugation, supernatant was subjected to the measurement of total protein content and MDA levels. MDA levels were detected by microplate reader at 532 nm and normalized to milligram protein.

### Statistical analysis

Data were presented as mean ± SD from at least 3 independent experiments. *P* values were evaluated by Student's two-tailed *t*-test using GraphPad Prism 7 software (GraphPad Software Inc., CA, USA). *P* < 0.05 was considered as statistically significant.

## Results

### Rhein inhibits PC cell growth by inducing a G1 phase cell cycle arrest

To investigate the effects of Rhein on cell growth, human PC cells (Panc-1 and MIAPaca-2) and the immortalized normal human pancreatic duct epithelial (HPDE) cell line were treated with varying concentrations (0-200 μM) of Rhein for 24 h and 48 h. CCK-8 assay results showed that Rhein inhibited cell viability in a dose- and time-dependent manner in Panc-1 and MIAPaca-2 cells, while HPDE cells were less sensitive to Rhein (Figure 1B). The respective inhibitory concentration (IC) 50 values at 24 and 48 h of Rhein treatment were approximately 89.9 μM and 76.3 μM in Panc-1 cells, 83.4 μM and 72.6 μM in MIAPaca-2 cells, and 157.1 μM and 150.4 μM in HPDE cells. Colony formation assays indicated that Rhein also inhibited the long-term survival ability of PC cells (Figure 1C).

To explore the mechanism of Rhein-induced growth inhibition in PC cells, cell cycle distribution was analyzed by flow cytometry. As shown in Figure 1D, Rhein caused G1 phase arrest of Panc-1 and MIAPaca-2 cells in a dose-dependent manner. Conversely, the percentage of HPDE cells in G1 phase was only slightly elevated under a high concentration of Rhein treatment. To understand the mechanism underlying the G1 phase arrest in Rhein-treated PC cells, we next used western blots to investigate the effect of Rhein on the expression levels of proteins that regulate the G1 phase transition. As shown in Figure 1E, Rhein treatment resulted in a dose-dependent decrease in CDK4, CDK6, cyclin D1, and cyclin E protein levels and an increase in p21 and p27 protein levels in PC cells. However, expression levels of these proteins only slightly changed in Rhein-treated HPDE cells. Taken together, these results indicate that Rhein can inhibit PC cell growth by inducing a G1 phase cell cycle arrest.

### Rhein induces PC cell apoptosis by activation of caspase cascades and the mitochondrial apoptosis pathway

**Figure 2:**
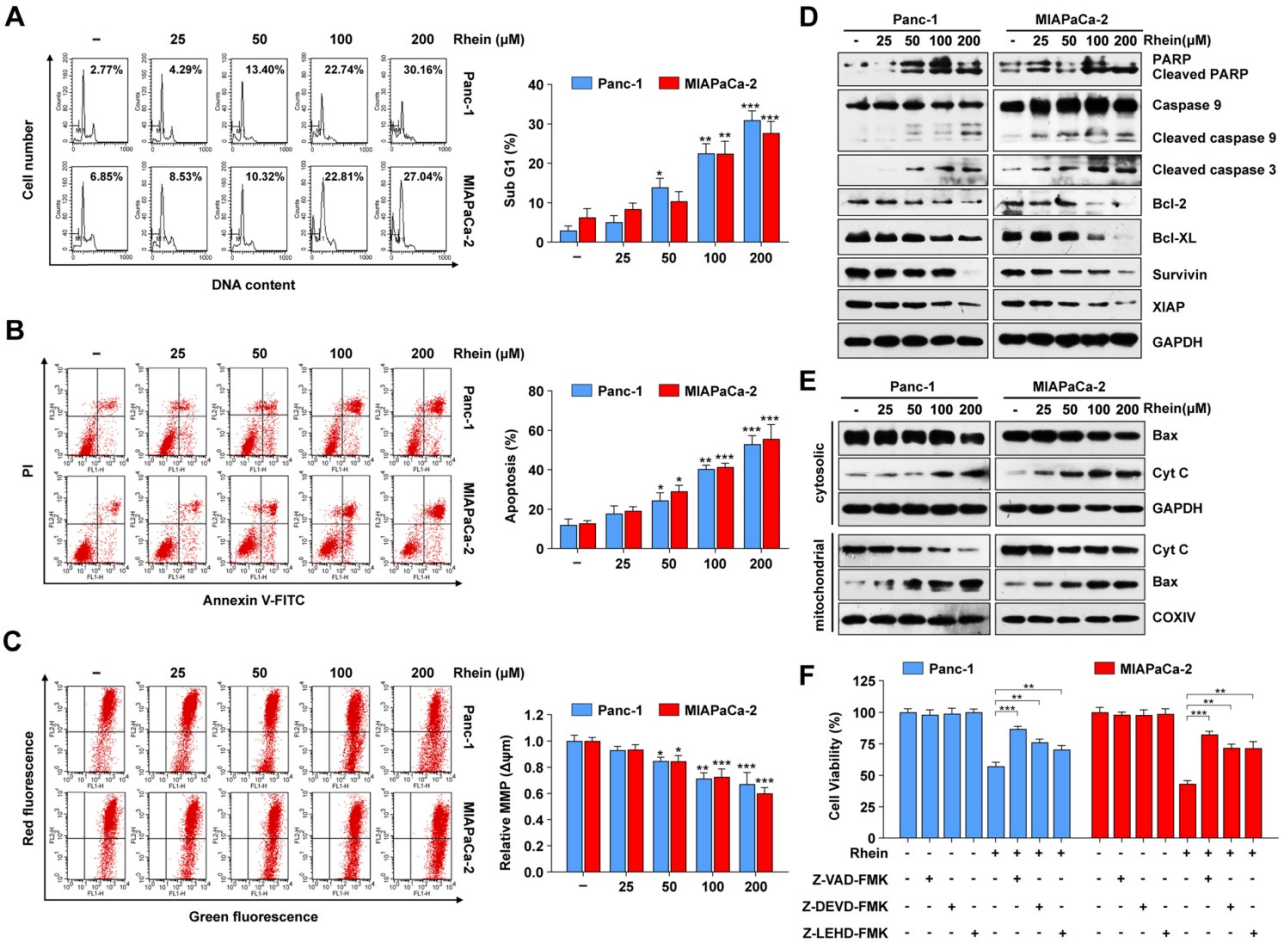
** Rhein induces PC cell apoptosis by activation of caspase cascades and the mitochondrial apoptosis pathway.** (A) Percentage of Panc-1 and MIAPaCa-2 cells in sub-G1 phase after 0-200 μM Rhein treatment for 24 h. Representative results are shown in the left panel. Each bar represents means ± SD from three independent experiments. *p < 0.05; **p < 0.01; ***p < 0.001. (B) Apoptosis rate of Panc-1 and MIAPaCa-2 cells were determined by Annexin V/PI double staining after 0-200 μM Rhein treatment for 24 h. Each bar represents means ± SD from three independent experiments. *p < 0.05; **p < 0.01; ***p < 0.001. (C) Panc-1 and MIAPaCa-2 cells were treated with indicated concentration of Rhein for 24 h. Mitochondrial membrane potential (Δ*ψ*m) was determined by JC-1 staining and flow cytometry analysis. Each bar represents means ± SD from three independent experiments. *p < 0.05; **p < 0.01; ***p < 0.001. (D) Western blot analysis of PARP, caspase-9, cleaved-caspase-3, Bcl-2 and IAP family proteins levels in Panc-1 and MIAPaCa-2 cells after 0-200 μM Rhein treatment for 24 h. GAPDH was used as loading control. Representative results of two independent experiments are shown. (E) Western blot analysis of Bax and cytochrome c (Cyt c) in mitochondria and cytoplasm of Panc-1 and MIAPaCa-2 cells after 0-200 μM treatment for 24 h. GAPDH or COXIV was used as loading control of cytoplasm or mitochondrial, respectively. (F) Panc-1 and MIAPaCa-2 cells were pre-treated or not with Z-VAD-FMK (50 μM), z-DEVD-FMK (75 μM) or z-LEHD-FMK (75 μM) for 1 h, followed by a 24 h treatment of 100 μM Rhein. Cell viability was measured by CCK-8 assay. Each bar represents means ± SD from three independent experiments. **p < 0.01; ***p < 0.001.

To explore whether Rhein has an effect on PC cell apoptosis, Panc-1 and MIAPaca-2 cells were treated with various concentrations of Rhein for 24 h and subjected to flow cytometry for sub-G1 phase cell analysis. As shown in Figure 2A, the percentages of Panc-1 and MIAPaca-2 cells in sub-G1 phase strikingly increased in a dose-dependent manner following Rhein treatment, which indicated an increase in the number of apoptotic cells. Next, we performed Annexin V/PI staining assays to ascertain if Rhein treatment elicited apoptosis. As shown in Figure 2B, the percentage of apoptotic Panc-1 and MIAPaca-2 cells increased in a dose-dependent manner after Rhein treatment. Considering the important role of mitochondria in cell survival and apoptosis 20, we analyzed the mitochondrial transmembrane potential (Δ*ψ*m) by JC-1 staining. Rhein treatment resulted in a dose-dependent loss of Δ*ψ*m, which is associated with mitochondria dysfunction and apoptosis (Figure 2C). Next, we investigated proteins that are closely related with the mitochondria-mediated apoptosis pathway using western blot analysis. In PC cells treated with Rhein, we observed a dose-dependent upregulation of the cleavage of caspase-9, caspase-3, and PARP, which indicated activation of the caspase cascade. In addition, the anti-apoptotic proteins Bcl-2, Bcl-XL, survivin, and XIAP were downregulated, suggesting that the mitochondrial pathway participates in Rhein-induced apoptosis (Figure 2D). Conversely, in HPDE cells, a lower apoptosis rate was detected, and cleaved PARP and cleaved caspase-3 were upregulated only under high concentrations of Rhein (Figure S1A and B). We also detected an accumulation of Bax in the mitochondria, together with a release of cytochrome c from the mitochondria to the cytosol, in Panc-1 and MIAPaca-2 cells after Rhein treatment. These observations suggest a dissipation of Δ*ψ*m and loss of mitochondrial function (Figure 2E). Interestingly, the pan-caspase inhibitor z-VAD-FMK strongly prevented PC cells from Rhein-induced cell death (Figure 2F). Moreover, z-DEVD-FMK and z-LEHD-FMK, inhibitors of caspase-3 and -9, respectively, both effectively inhibited the death induced by Rhein, suggesting that Rhein-induced cell apoptosis was caspase-dependent. Together, these results suggested that Rhein can induce caspase-dependent mitochondrial apoptosis in PC cells.

### Rhein induces PC cell apoptosis through inactivation of the PI3K/AKT pathway

**Figure 3:**
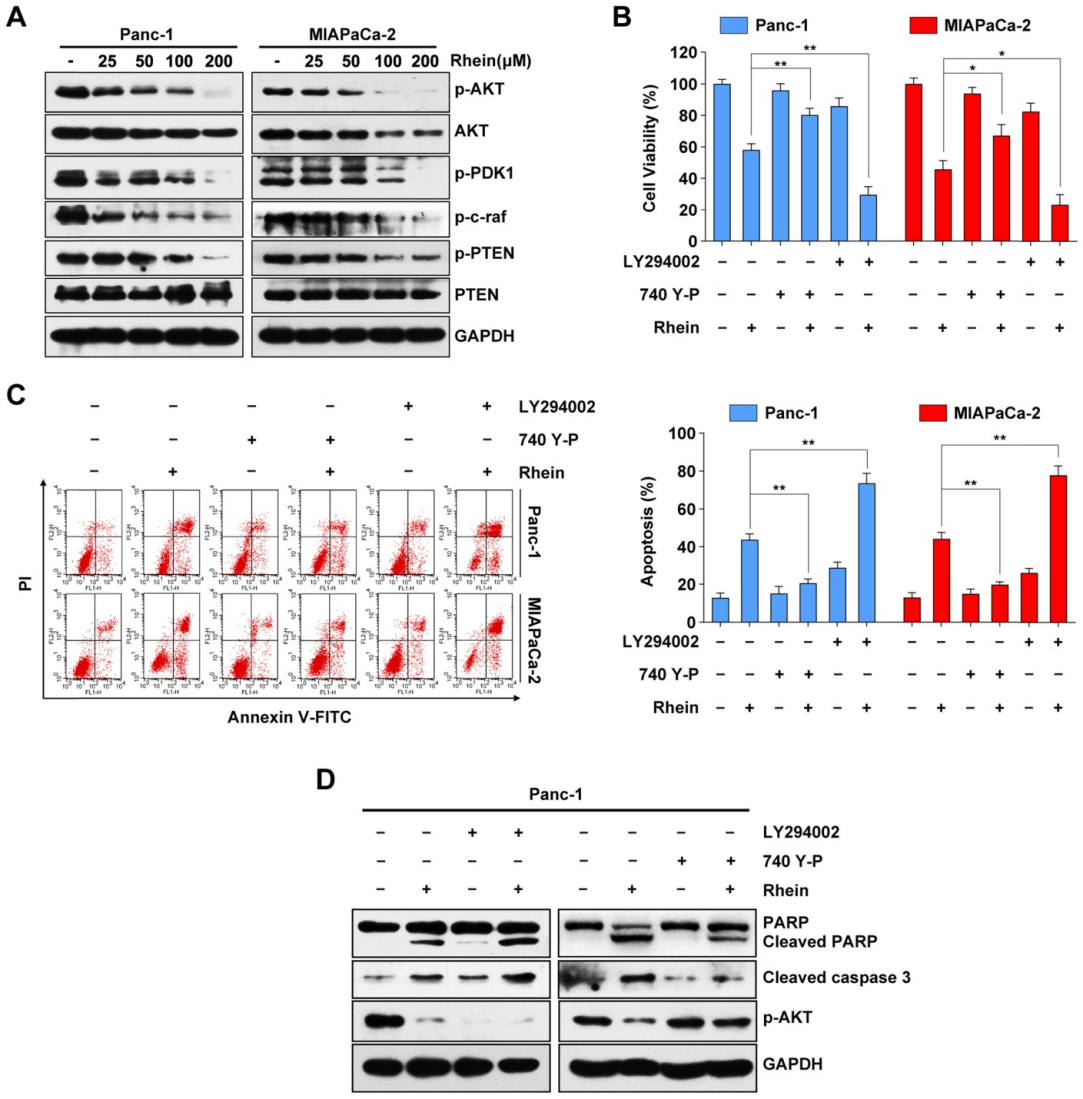
** Rhein induces PC cells apoptosis through inactivation of the PI3K/AKT pathway.** (A) Western blot analysis of PI3K/AKT pathway proteins levels in Panc-1 and MIAPaCa-2 cells after 0-200 μM Rhein treatment for 24 h. (B) Panc-1 and MIAPaCa-2 cells were pre-treated or not with LY294002 or 740Y-P, with or without 100 μM Rhein treatment for 24 h. Cell viability was measured by CCK-8 assay. Each bar represents means ± SD from three independent experiments. *p < 0.05; **p < 0.01. (C) Apoptosis rates were determined by Annexin V/PI double staining after cells were treated as described in (B). Each bar represents means ± SD from three independent experiments. **p < 0.01. (D) Western blot analysis of PARP, cleaved-caspase-3 and phospho-AKT in Panc-1 cells treated as described in (B).

Previous studies have reported that the PI3K/AKT signaling pathway plays an important role in cell proliferation and survival through downstream signal transduction cascades in PC 21, 22. To determine whether Rhein has an impact on the PI3K/AKT pathway, we used western blots to investigate expression levels of key proteins of this pathway following Rhein treatment of Panc-1 and MIAPaCa-2 cells. As shown in Figure 3A, we observed a decrease in the phosphorylated levels of AKT, PDK1, and c-raf after Rhein treatment. Moreover, the levels of phosphorylated PTEN (inactive PTEN) were reduced upon Rhein treatment without a significant change in levels of total PTEN protein. Next, Panc-1 and MIAPaCa-2 cells were pre-treated with a PI3K/AKT-specific inhibitor (LY294002) or activator (740Y-P). Pre-treatment with LY294002 or 740Y-P markedly enhanced or attenuated the apoptosis of PC cells induced by Rhein, respectively (Figure 3B and 3C). Consistent with these results, the levels of cleaved PARP and caspase-3 were increased in Rhein-treated Panc-1 cells after pre-treatment with LY294002. However, the opposite effect was observed after 740Y-P pre-treatment of these cells (Figure 3D). These results confirm that the PI3K/AKT signaling pathway is involved in Rhein-induced PC cell apoptosis.

Another important signaling pathway in cell proliferation and survival is the MAPK pathway. Western blot results showed that the phosphorylation levels of JNK, p38 and ERK1/2 were increased after Rhein treatment (Figure S2A). However, pre-treatment with SP600125, SB203580, and PD98059, which are respective inhibitors of JNK, p38 and ERK1/2, had little effect on the cell death induced by Rhein treatment (Figure S2B). Overall, these results show that Rhein-induced apoptosis in PC cells is associated with the PI3K/AKT pathway, but not the MAPK pathway.

### Rhein synergistically enhances the therapeutic effects of oxaliplatin in PC cells via suppression of PI3K/AKT signaling

**Figure 4:**
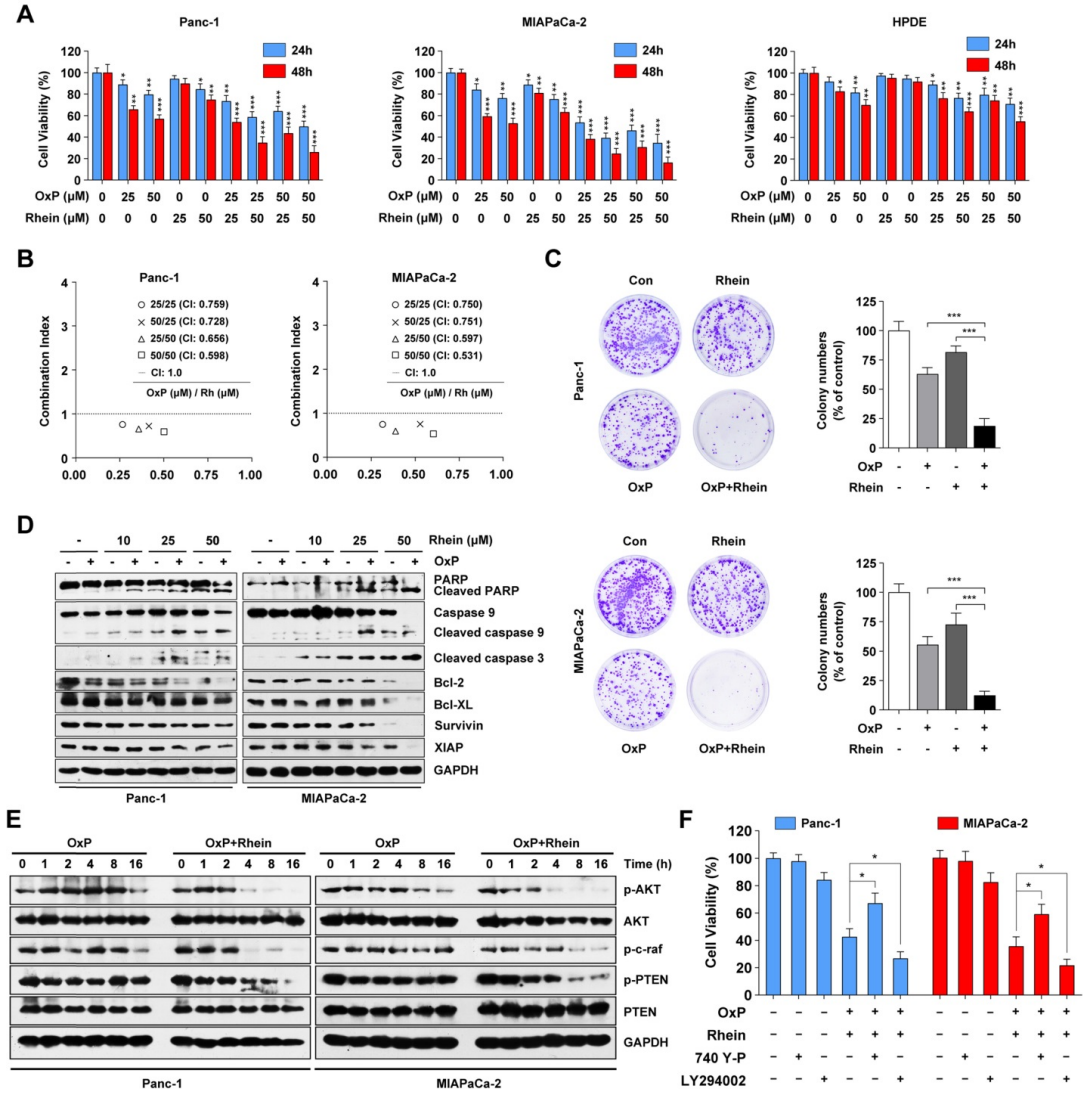
** Rhein synergistically enhances the therapeutic effects of oxaliplatin in PC cells via suppression of PI3K/AKT signaling.** (A) Panc-1, MIAPaCa-2 and HPDE cells were treated with indicated concentration of oxaliplatin and Rhein for 24 or 48 h. CCK-8 assays were performed to determine the cell viability. (B) Combination Index (CI) values for oxaliplatin and Rhein in Panc-1 and MIAPaCa-2 cells were constructed by CalcuSyn software. CI < 1.0 indicates synergistic effect. (C) Panc-1 and MIAPaCa-2 cells were treated with oxaliplatin (25 μM) or Rhein (50 μM) alone or in combination for 24 h and allowed to form colonies for 14 days. Representative images are shown. Each bar represents means ± SD from three independent experiments. ***p < 0.001. (D) Western blot analysis of PARP, caspase-9, cleaved-caspase-3, Bcl-2 and IAP family proteins levels in Panc-1 and MIAPaCa-2 cells after treated with indicated concentration of oxaliplatin and Rhein. GAPDH was used as loading control. (E) Western blot analysis of PI3K/AKT pathway proteins treated with oxaliplatin (25 μM) or combination of oxaliplatin (25 μM) and Rhein 50 μM) for indicated times in Panc-1 or MIAPaCa-2 cells. GAPDH was used as loading control. (F) Panc-1 and MIAPaCa-2 cells were pretreated with 740Y-P or LY294002 for 1 h, followed by combination of oxaliplatin (25 μM) and Rhein (50 μM) treatment for 24 h. Cell viability was measured by CCK-8 assay. Each bar represents means ± SD from three independent experiments. *p < 0.05.

Oxaliplatin, a commonly used chemotherapeutic agent available for the treatment of advanced PC, is not very effective on its own and is associated with systemic toxicity and chemoresistance 23. Because aberrant activation of the PI3K/AKT pathway has been shown to promote the drug resistance ability of PC cells 24, we next evaluated whether Rhein can potentiate the effect of oxaliplatin in these cell lines. As shown in Figure 4A, CCK-8 assays showed that the combination treatment of oxaliplatin and Rhein enhanced the growth inhibitory effect on Panc-1 and MIAPaCa-2 cells compared with either agent alone. Conversely, HPDE cells were less sensitive to the combination treatment. To explore whether oxaliplatin and Rhein had a synergistic or only additive effect, the combination index (CI) of the two drugs was evaluated by CalcuSyn software. CI<1, CI=1, and CI>1 indicate synergistic, additive, and antagonistic effects, respectively. As shown in Figure 4B, the CI values were lower than 1 in both Panc-1 and MIAPaCa-2 cells under different concentrations of oxaliplatin and Rhein treatment, indicating that the two drugs had a synergistic effect. Colony formation assays further confirmed this conclusion, as the colony numbers in the combined treatment group were significantly lower than the control or single drug groups (Figure 4C).

We next examined whether the effect of the combination treatment involved the enhancement of apoptosis. Western blot assays revealed that combined treatment of these cell lines with oxaliplatin and Rhein resulted in a dramatic increase in the cleavage of PARP, caspase-9, and caspase-3, as well as a decrease in the levels of anti-apoptotic proteins Bcl-2, Bcl-XL, survivin, and XIAP, compared with either agent alone (Figure 4D). From these data, we inferred that Rhein potentiates the cytotoxic effect of oxaliplatin by reducing cell viability and promoting apoptosis in PC cells.

To investigate whether the PI3K/AKT signaling pathway is involved in the effect of the combined treatment of oxaliplatin and Rhein in PC cells, we investigated the key proteins in this pathway following the combination and control treatments. Western blot results suggested that phosphorylated AKT was activated by oxaliplatin in Panc-1 cells. Conversely, Rhein plus oxaliplatin treatment remarkably decreased the phosphorylated levels of AKT, c-raf, and PTEN in both Panc-1 and MIAPaCa-2 cells compared with oxaliplatin treatment alone (Figure 4E). The PI3K/AKT inhibitor LY294002 and activator 740Y-P were used to determine whether this pathway was involved in combination treatment-induced apoptosis. As shown in Figure 4F, pre-treatment with 740Y-P attenuated the apoptosis induced by the combination treatment, while LY294002 pre-treatment potently enhanced the combination treatment-induced cell death. Thus, we conclude that the combination treatment of oxaliplatin and Rhein induces apoptosis of PC cells via inhibition of the PI3K/AKT pathway.

### ROS generation is critically involved in apoptosis induced by the combination of oxaliplatin and Rhein

**Figure 5:**
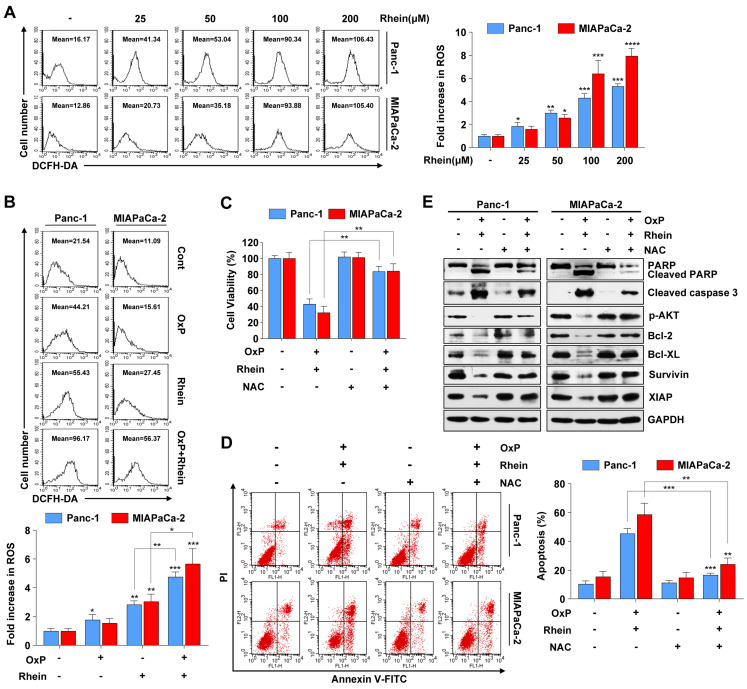
** ROS generation is critically involved in apoptosis induced by the combination of oxaliplatin and Rhein.** (A) Panc-1 and MIAPaCa-2 cells were treated with 0-200 μM Rhein for 12 h and incubated with 10 μM DCFH-DA for flow cytometry analysis. Representative results are shown in the left panel. Each bar represents means ± SD from three independent experiments. *p < 0.05; **p < 0.01; ***p < 0.001. (B) Panc-1 and MIAPaCa-2 cells were treated with oxaliplatin (25 μM) or Rhein (50 μM) alone or in combination for 12 h and incubated with 10 μM DCFH-DA for flow cytometry analysis. Each bar represents means ± SD from three independent experiments. *p < 0.05; **p < 0.01; ***p < 0.001. (C) Panc-1 and MIAPaCa-2 cells were pretreated or not with NAC (5 mM) for 1 h, followed by combined treatment of oxaliplatin (25 μM) and Rhein (50 μM). Cell viability was measured by CCK-8 assay. Each bar represents means ± SD from three independent experiments. **p < 0.01. (D) Panc-1 and MIAPaCa-2 cells were treated as described in (C) and apoptosis rates were determined by Annexin V/PI double staining. Each bar represents means ± SD from three independent experiments. **p < 0.01; ***p < 0.001. (E) Western blot analysis of PARP, cleaved-caspase-3, phospho-AKT and Bcl-2 family proteins in Panc-1 and MIAPaCa-2 cells treated as described in (C). GAPDH was used as loading control.

ROS is an important mediator of oxidative stress and can induce apoptosis if produced in excessive amounts 25. Recent studies have demonstrated that various anti-cancer agents induce cancer cell apoptosis through ROS generation 26, 27. To explore whether Rhein has an influence on cellular ROS levels, we used flow cytometry to detect ROS after Rhein treatment. As shown in Figure 5A, cellular ROS levels were elevated in a dose-dependent manner after Rhein treatment. Furthermore, the combined treatment of oxaliplatin and Rhein significantly increased ROS levels compared with treatment with either drug alone (Figure 5B). Next, the ROS scavenger NAC was used to investigate whether the combination treatment-induced apoptosis involved excessive generation of ROS. Pre-treatment of Panc-1 and MIAPaCa-2 cells with NAC for 1 h effectively reversed the cell death induced by the combination treatment (Figure 5C). Flow cytometry also showed that NAC reversed the combination treatment-induced apoptosis (Figure 5D). Additionally, we investigated the change of apoptotic protein levels with NAC pre-treatment. Consistent with our hypothesis, NAC pre-treatment dramatically attenuated the combination treatment-induced cleavage of PARP and caspase-3, while it recovered the protein levels of phosphorylated AKT, Bcl-2, Bcl-XL, survivin, and XIAP (Figure 5E). Taken together, these results suggest that elevation of ROS levels is a critical event in apoptosis induced by the combination of oxaliplatin and Rhein.

### Combination of oxaliplatin and Rhein synergistically inhibits tumor growth and induces PC cell apoptosis in a murine xenograft model

**Figure 6:**
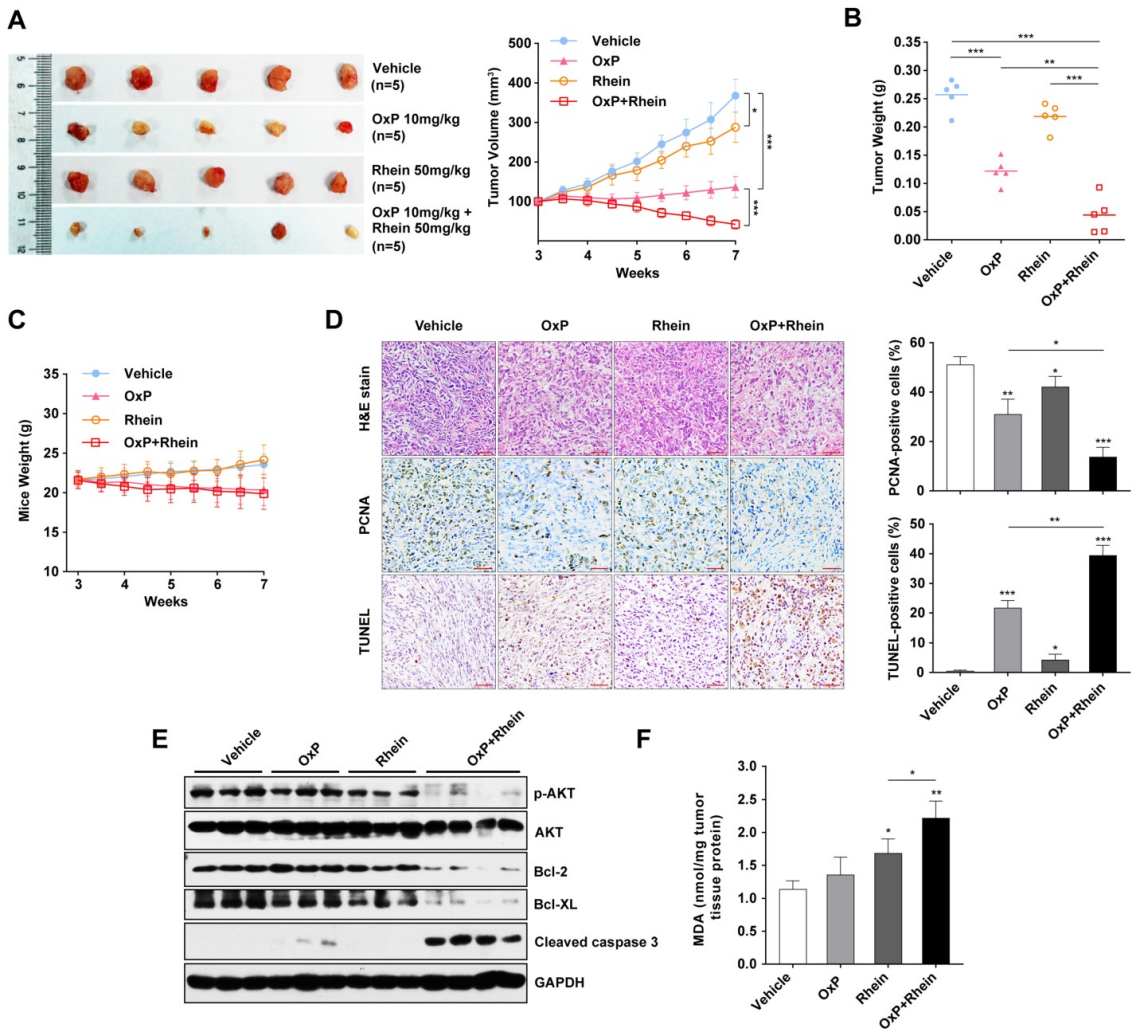
** Combination of oxaliplatin and Rhein showed synergistic antitumor activity in a murine xenograft model.** (A) Tumors of mice from each group on week 7 after tumor implantation. Tumor volume of each group were measured twice a week from week 3 after tumor implantation. *t*-tests were used to evaluate the statistical difference between groups. *p < 0.05; ***p < 0.001. (B) Tumor weight of each group on week 7 collected immediately after sacrificing the animals. Average tumor weight for each group was calculated. **p < 0.01; ***p < 0.001. (C) Body weight of mice were measured twice a week from week 3 after tumor implantation (D) H&E and immunohistochemical analysis for PCNA and TUNEL analysis of tumor xenografts harvested from mice. Scale bar: 400μm. Statistical analysis of positive cells are shown in the right panel. *p < 0.05; **p < 0.01; ***p < 0.001. (E) Western blot analysis of levels of phospho-AKT, AKT, Bcl-2, Bcl-XL and cleaved-caspase-3 from tumor xenograft proteins. GAPDH was used as loading control. (F) MDA levels of tissue proteins were analyzed. Each bar represents means ± SD in each group. *p < 0.05; **p < 0.01.

To explore the anti-tumor activity of combination of oxaliplatin and Rhein *in vivo*, we investigated whether tumor development was inhibited by the combination treatment in a murine xenograft model. The growth of tumor xenografts was inhibited by treatment with oxaliplatin or Rhein alone, and a synergistic anti-tumor effect was achieved in the combined treatment group (Figure 6A). Mean tumor weights were also remarkably decreased in the combination group compared with other groups (Figure 6B). Moreover, mice receiving oxaliplatin appeared to be sick with loss of appetite and weight loss, but no marked addition of toxicity in terms of progressive weight loss was observed in the combination group (Figure 6C).

Immunohistochemistry was also performed to evaluate the proliferation and apoptosis of xenograft tumors. We found a significant decrease in proliferating cell nuclear antigen (PCNA)-positive cells and an increase in TUNEL-positive cells in the combined treatment group, which indicated the suppression of proliferation and activation of apoptosis (Figure 6D). Western blot analysis of xenograft tumors was also performed, which revealed increased levels of cleaved caspase-3 and decreased phosphorylated AKT, Bcl-2, and Bcl-XL in the combined treatment group (Figure 6E). These findings suggest that the PI3K/AKT signaling pathway was inhibited and apoptosis was activated in the tumor tissues. Furthermore, the MDA assay was used to detect ROS-mediated injury in tissues. MDA is a product of lipid peroxidation and is used for quantifying oxidative stresss 28. We found a significantly higher MDA level in the combined treatment group compared with the single treatment groups, which indicated an increase in ROS-mediated injury (Figure 6F). Collectively, these results clearly demonstrated that the combination of oxaliplatin with Rhein had synergistic anti-tumor activity in human PC xenograft models.

## Discussion

In addition to the single drug treatments for PC, such as the deoxycytidine nucleoside analog gemcitabine, progress has been made in developing and identifying effective combined chemotherapies for PC in recent years 29. Various natural products that enhance the anti-tumor activity and alleviate the side effects of conventional chemotherapy drugs have gained much attention. In the present study, we demonstrated that Rhein, a main constituent of the Chinese herb rhubarb, inhibits the growth and proliferation of PC cells through inducing a G1 phase cell cycle arrest. Treatment of PC cells with Rhein also resulted in caspase-dependent mitochondrial apoptosis through inactivation of the PI3K/AKT pathway, yet normal HPDE cells were less sensitive to such treatment. In addition, low-dose Rhein potentiated the growth inhibition and apoptosis-inducing effects of oxaliplatin in PC cells through increased generation of intracellular ROS. This was the specific event upstream of the inhibition of PI3K/AKT signaling and induction of apoptosis. Furthermore, combination treatment of oxaliplatin and Rhein synergistically reduced tumor growth without additional toxicity in PC xenografts. To the best of our knowledge, this is the first study showing that Rhein synergistically enhances the therapeutic effects of oxaliplatin both *in vitro* and *in vivo* in PC.

Accumulating evidence has demonstrated that cell cycle regulators are aberrantly expressed in various human cancers and thus targeting the cell cycle is an effective strategy against cancer cell proliferation 30. Cyclins and CDKs are critical regulators of cell cycle progression. Cyclin D1 activates CDK4 and CDK6, which promotes cells into S phase together with cyclin E. In contrast, the KIP/CIP proteins p21 and p27 act as negative regulators by inhibiting cyclin-CDK complexes 31. Rhein can reportedly induce G1 phase cell cycle arrest in HepaRG, HepG2, and Huh7 cells 32, 33. Likewise, we found a significant G1 phase cell cycle arrest, downregulation of CDKs and cyclins, and upregulation of p21 and p27 in Panc-1 and MIAPaca-2 cells after Rhein treatment, suggesting the inhibitory effect of Rhein on the cell cycle in PC.

The mitochondrial apoptosis pathway, also called the intrinsic apoptosis pathway, is regulated cell death triggered by mitochondrial outer membrane permeabilization (MOMP) that results in the loss of Δ*ψ*m and the release of apoptogenic factors such as cytochrome c from the mitochondria to cytoplasm. This subsequently activates the caspase cascade and leads to cell death 34. This process is regulated by a series of proteins, including pro-apoptotic members of the Bcl-2 family such as Bax, Bak and Bid, and anti-apoptotic members of the same family such as Bcl-2, Bcl-XL. Activation of the caspase cascade can be inhibited by IAP family proteins, including XIAP and survivin 35, 36. In PC cells treated with Rhein, we observed a decrease in Δ*ψ*m, which indicated the dissipation of mitochondrial membrane permeabilization. Moreover, activation of caspase-9, caspase-3, and PARP, as well as the release of cytochrome c, further suggested the pivotal role of the mitochondrial apoptosis pathway in Rhein-induced PC cell apoptosis. Additionally, expression levels of the pro-apoptotic protein Bax increased, while levels of the anti-apoptotic proteins Bcl-2, Bcl-XL, survivin, and XIAP decreased, indicating the participation of Bcl-2 and IAP family proteins in Rhein-mediated apoptosis of PC cells. The effects of Rhein on apoptosis-related proteins are consistent with a previous study 19.

The PI3K/AKT signaling pathway is considered the “survival pathway” for its important role in cell differentiation, proliferation, and survival 37. Previous studies have demonstrated that AKT is upregulated in various malignant tumors, including gastric, colon, breast, and ovarian tumors, as well as PC 38. Aberrant activation of AKT has been found to be associated with tumorigenesis and drug resistance of cancer 39. Previous studies have shown the diverse influence of Rhein on AKT. Rhein was found to promote the activation of AKT in intestinal epithelial cells, while inhibiting its activation in non-small-cell lung cancer, breast cancer, and liver cancer cells 16, 40, 41. Our results showed that the PI3K/AKT pathway was inhibited after Rhein or combined drug treatment of PC cells, as reflected by the downregulation of phosphorylated levels of AKT, PDK1, c-raf, and PTEN. Moreover, pre-treatment with the PI3K/AKT-specific inhibitor LY294002 enhanced Rhein alone or combination treatment-induced apoptosis. Conversely, pre-treatment with activator 740Y-P partly rescued cells from death, indicating that Rhein alone or the combined treatment executed anti-tumor effects in an AKT-dependent manner. Recent studies have shown that MAPK pathways are also involved in Rhein-induced cell death 14, 42. In our study, we found that Rhein treatment also activated JNK, p38, and ERK1/2. However, no significant reverse effect was observed in cells pre-treated with various MAPK inhibitors, indicating that MAPK signaling was not involved in the apoptosis induced by Rhein in human PC cells.

Compared with normal cells, cancer cells have higher levels of ROS because of their enhanced metabolism and persistent pro-oxidative state, which leads to intrinsic oxidative stress 43. ROS can induce DNA damage and increase the mutation rate, contributing to tumorigenesis. A high level of ROS can also increase the susceptibility of tumor cells to oxidative stress-induced cell death, which could be a potential strategy for cancer therapy 44. ROS has been found to induce endoplasmic reticulum stress and promote apoptosis of PC cells 45. Previous studies have demonstrated that oxaliplatin induces intracellular ROS production and accumulation, which contributes to its anti-cancer activity 28. In the present study, we found an elevation in the ROS level in Rhein-treated PC cells. Furthermore, the ROS level was higher in cells following the combined treatment of oxaliplatin and Rhein compared with cells treated with each drug alone, indicating the synergistic effect of Rhein and oxaliplatin on ROS induction. More importantly, pre-treatment of cells with the antioxidant NAC dramatically attenuated the cleavage of PARP and caspase-3, recovered the protein levels of Bcl-2, Bcl-XL, survivin, and XIAP, and protected PC cells from apoptosis after treatment with Rhein plus oxaliplatin. Furthermore, we observed that the level of phosphorylated AKT was restored in the NAC-treated group, indicating that ROS is an upstream regulator of PI3K/AKT inactivation in the combined treatment group. Similar to our findings, ROS has also been found to inhibit the PI3K/AKT pathway in various tumor cells treated with natural anti-tumor drugs 46, 47.

In conclusion, the present study revealed that Rhein can inhibit proliferation and subsequently induce apoptosis through inactivation of the PI3K/AKT pathway in PC cells. In addition, our data demonstrated a synergistic effect of Rhein and oxaliplatin against PC cells *in vitro* and in tumor xenograft models by the induction of excessive ROS accumulation and repressing AKT activation. Our results showed that ROS is the specific event upstream of the AKT inhibition and apoptosis induction. Therefore, these findings indicate that the combination of Rhein and oxaliplatin could be a novel strategy to overcome drug resistance in the chemotherapeutic treatment of PC.

## Supplementary Material

Supplementary figures.

## Abbreviations

AKT: protein kinase B
Bcl-2: B-cell lymphoma 2
CCK-8: Cell counting kit-8
CDK: cyclin dependent kinase
CI: combination index
EGFR: epidermal growth factor receptor
ERK: extracellular regulated protein kinases
IC50: half-maximal inhibitory concentration
JNK: c-Jun N-terminal kinase
MAPK: mitogen-activated protein kinase
MDA: Malondialdehyde
MOMP: Mitochondrial outer membrane permeabilization
NAC: N-acetyl-L-cysteine
NF-κB: nuclear factor-kappa B
p-AKT: phosphorylated AKT
PARP: poly-ADP-ribose polymerase
PC: Pancreatic cancer
PCNA: proliferating cell nuclear antigen
PDK1: 1, 3 phosphoinositide‐dependent protein kinase 1
PI: propidium iodide
PI3K: phosphoinositide‐3‐kinase
PTEN: phosphatase and tensin homologue deleted on chromosome 10
ROS: Reactive oxygen species
STAT3: signal transducer and activator of transcription 3
TUNEL: TdT-mediated dUTP Nick-End Labeling
XIAP: X-linked inhibitor of apoptosis protein
