# Supplementary material for: Inhibition of PI3K/AKT signaling via ROS regulation is involved in Rhein-induced apoptosis and enhancement of oxaliplatin sensitivity in pancreatic cancer cells
Source: Int J Biol Sci. 2021 Jan 15;17(2):589–602. doi: 10.7150/ijbs.49514 (PMC7893580; doi:10.7150/ijbs.49514)
Supplement: Supplementary file 1 — Supplementary figures. [file ijbsv17p0589s1.pdf]

Supplementary Materials

Antibodies and reagents

SAPK/JNK (#9252), phospho-SAPK/JNK (Thr183/Tyr185) (#9255), p38 (#8690), phospho-p38 (Thr180/Tyr182) (#4511), ERK1/2 (#4695) and phospho-ERK1/2 (Thr202/Tyr204) (#8544) antibodies were purchased from Cell Signaling Technology (Beverly, MA, USA). SB203580 (HY-10256), PD98059 (HY-12028) and SP600125 (HY-12041) were purchased from MCE (Monmouth, NJ, USA).

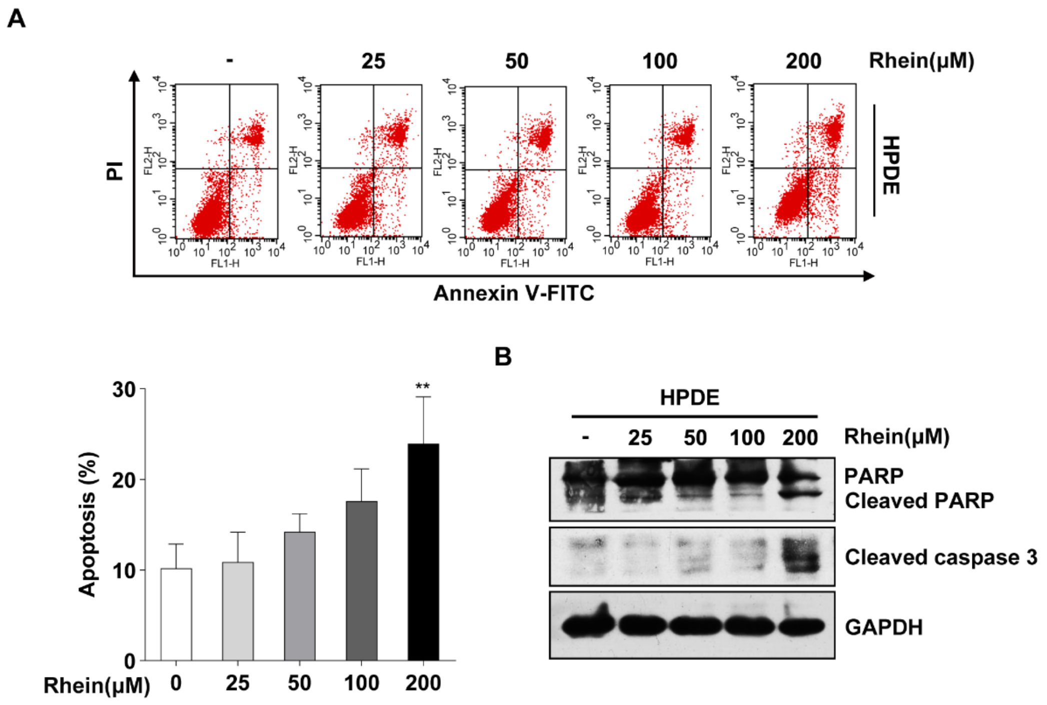

SUPPLEMENTARY FIGURE S1 Effects of Rhein on the apoptosis of HPDE cells. (A) Apoptosis rate of HPDE cells were determined by Annexin V/PI double staining after 0-200 μM Rhein treatment for 24 h. Each bar represents means ± SD from three independent experiments. $^{**}p < 0.01$. **(B)** Western blot analysis of PARP and cleaved-caspase-3 proteins levels in HPDE cells after 0-200 μM Rhein treatment. GAPDH was used as loading control.

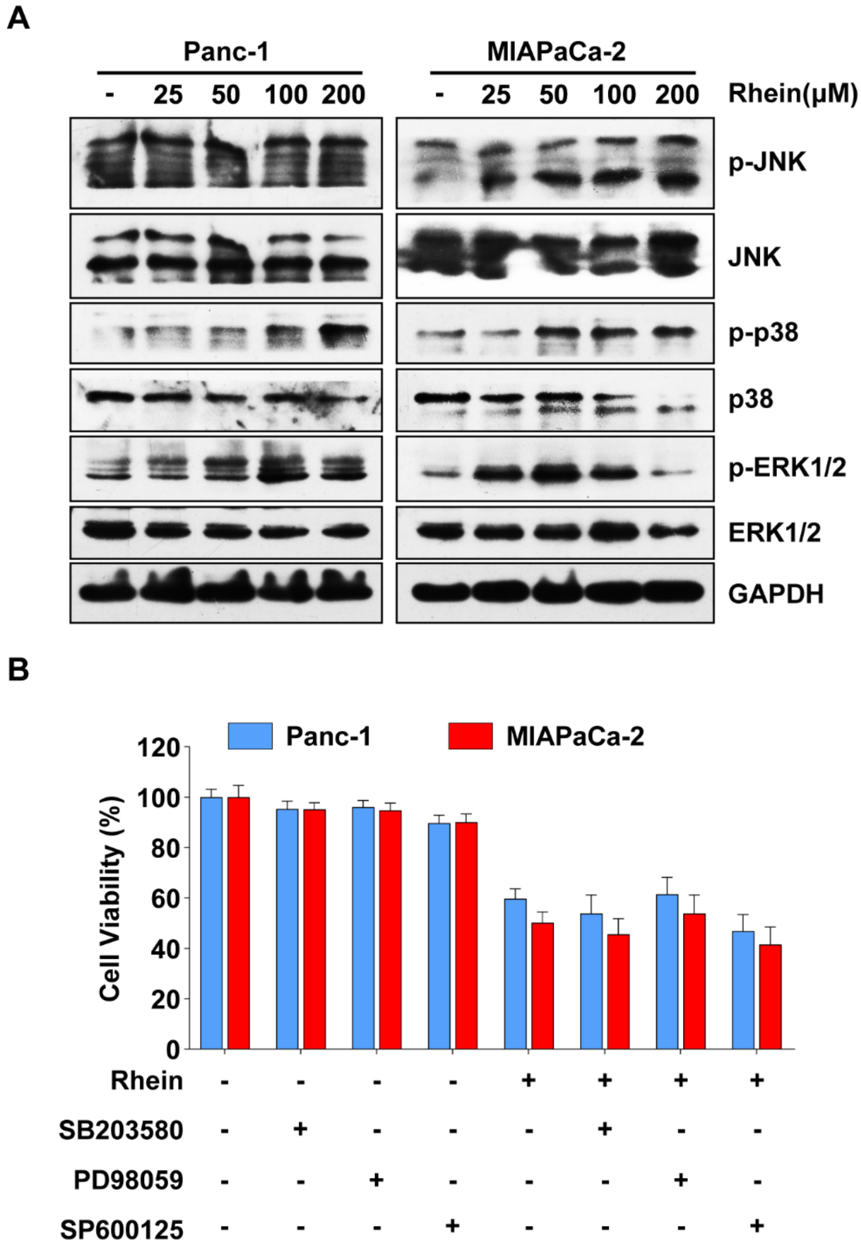

SUPPLEMENTARY FIGURE S2 MAPKs signaling pathway was not relevant in Rhein-induced apoptosis. **(A)** Western blot analysis of MAPKs signaling proteins levels in Panc-1 and MIAPaCa-2 cells after 0-200 μM Rhein treatment. **(B)** Panc-1 and MIAPaCa-2 cells were pretreated with SB203580 (25 μM), PD98059 (25 μM), or SP600125 (25 μM) for 1 h followed by Rhein (100 μM) treatment for 24 h. Cell viability was measured by CCK-8 assay. Each bar represents means ± SD from three independent experiments.
